# Determination of the Color Change of Various Esthetic Monolithic Monochromatic Computer-Aided Design/Computer-Aided Manufacturing Materials

**DOI:** 10.3390/ma17133160

**Published:** 2024-06-27

**Authors:** Katarina Bauer, Andreja Carek, Ljerka Slokar Benić, Tomislav Badel

**Affiliations:** 1 School of Dental Medicine, University of Zagreb, Ivana Gundulića 5, 10000 Zagreb, Croatia; katarina.sturman@gmail.com (K.B.); acarek@sfzg.unizg.hr (A.C.); badel@sfzg.unizg.hr (T.B.); 2 Faculty of Metallurgy, University of Zagreb, Aleja narodnih heroja 3, 44000 Sisak, Croatia

**Keywords:** color stability, esthetic dental materials, monolithic monochromatic materials, CAD/CAM materials, thermocycling, staining solution

## Abstract

Dental technology has developed materials for prosthetics that are very similar to natural teeth and offer a good balance between durability and esthetics; however, some of these materials are not very color-stable under the influence of external factors. Therefore, the aim of this study is to determine and compare the color change (∆E_00_) of different esthetic monolithic monochromatic CAD/CAM materials after they have been thermocycled and treated with staining solutions. The color parameters were determined using a spectrophotometer on a white and black background. Five CAD/CAM materials were used for this study—CAD/CAM nanoceramic (GC Cerasmart270), CAD/CAM lithium disilicate ceramic (IPS e.max CAD), CAD/CAM polymer (Telio CAD), CAD/CAM composite (Tetric CAD) and CAD/CAM polymer-infiltrated ceramic (Vita Enamic). The test specimens produced were divided into groups and were thermocycled in distilled water. They were then stored for four weeks at 37 °C in either distilled water as a control liquid, black tea, instant coffee or red wine. The aim is also to evaluate the color changes as a function of the exposure time of the staining solutions. The results obtained were analyzed statistically. All CAD/CAM materials tend to discolor to varying degrees. Among the factors contributing to discoloration, red wine proved to be the most significant influencing factor. The conclusion from the results is that the color change is influenced by the type of material, the staining solution, the sample thickness, the color background and the aging time.

## 1. Introduction

Modern times have brought many new technologies and materials to the world of dentistry. The durability and strength of dental restorations, as well as the field of dental prosthetics, have made significant advances in dental materials and technology to meet the increased esthetic expectations and demands of patients. The increased demand for improved esthetics has led to an increase in the use of esthetic materials. With the development of CAD/CAM technology, the field of metal-free prosthetic restorations has been significantly expanded and improved. A fundamental goal in the field of esthetic dentistry is to produce restorations that closely resemble the appearance of natural teeth. Esthetic materials are characterized by good optical properties and biocompatibility, making them particularly suitable for highly esthetic restorations [1,2,3]. Although advances have been made in dental restorative materials, including improvements in biocompatibility and improved mechanical and optical properties, the success of esthetic restorations depends primarily on the selection of the appropriate material.

Color stability is a key clinical feature for all dental materials. Any deviation from the original standard shade indicates the aging or deterioration of the material. The change in color with prolonged clinical use is attributed to various factors including stain accumulation, dissolution of material components, water absorption and surface roughness. Beverages such as tea, coffee, wine, acidic soft drinks and other artificial food colors can affect discoloration.

Polymers are widely used in dentistry, including polymethyl methacrylate (PMMA). CAD/CAM PMMA is used to fabricate longer-lasting temporary restorations due to its superior mechanical properties, flexural hardness and modulus of elasticity [3,4,5,6,7]. Composite materials are known to be inferior to ceramics in terms of strength and fracture resistance. They consist of three main components—an organic resin matrix, inorganic filler particles and a binder. CAD/CAM materials that are polymerized under controlled conditions at high temperatures and under pressure have better physical and mechanical properties. The color stability of CAD/CAM composites is higher than that of direct composites. In a study by Quek et al. comparing direct, indirect and CAD/CAM composites, it was found that the color of the mentioned materials (ΔE) changed after one week of contact with colored liquids, with direct and indirect composites having a higher ΔE value than CAD/CAM composites [8].

The most widely used material of choice with excellent esthetic properties is still glass–ceramic. Glass–ceramics have better optical and mechanical properties, with lithium disilicate ceramics being the most commonly used. IPS e.max CAD shows better color stability than the lithium disilicate ceramic IPS e.max press after thermocycling and exposure to colored liquids, while the zirconia ceramic shows the same stability as IPS e.max CAD [7,8]. Hybrid ceramics were developed to utilize the good properties of ceramics and polymers and combine them in a material with improved properties. In particular, the modulus of elasticity, strength and hardness of the material were adapted to obtain a material that resembles the properties of a natural tooth [9,10]. 

Hybrids are viewed as the integration of polymers into porous ceramic structures. These hybrids come in two main types based on their microstructure—dispersed fillers and polymer-infiltrated ceramic networks (PICNs). In PICNs, also referred to as resin interpenetrating network ceramic, the ceramic and polymer components create a cohesive three-dimensional interconnected framework. Vita Enamic exemplifies PICNs, whereas Cerasmart represents a resin with dispersed fillers [11,12,13]. In dental rehabilitation, the importance of color stability in dental materials is emphasized for esthetic reasons. Various foods and beverages have the potential to stain dental restorations upon contact, potentially leading to esthetic flaws. Compared to ceramics, composites have a significantly greater potential for discoloration [14].

The daily use of various esthetic materials produced with CAD/CAM technology raises the question of how these materials react to external influences that affect their color change. Today’s high consumption of foods and beverages rich in natural and synthetic colorants can lead to the unsatisfactory esthetic performance of dental materials [9]. The discoloration of materials is a process that is influenced by several external and internal factors. The consumption of pigmented foods and beverages, poor oral hygiene, the use of colored chlorhexidine-based mouthwashes and smoking are among the extrinsic discoloration factors. Internal colors depend on the composition of the material and the size of the particles, as well as the absorption of water [10,11,12,13,14].

Esthetics are becoming increasingly important. Therefore, it is important to know the optical properties of the different CAD/CAM materials in order to facilitate the selection of the right material and thus contribute to a more successful and durable fixed prosthetic work. Previous research has mainly focused on testing the mechanical properties of various restorative materials, while there have been few studies on the optical properties. In these studies, the optical properties of CAD/CAM composites are usually compared with those of direct composites [2,15,16,17], but not with those of hybrid and polymer materials. Both Nogueira et al. [18] and Bona et al. [15] investigated the optical properties, and Kanat-Erturk [19] investigated the color stability of CAD/CAM ceramics. Pecho et al. investigated the optical properties of esthetic resin composite restorative systems [20]. Dinelli et al. studied how coloring agents effect the optical properties of composite resins [21]. Elsaka et al. evaluated the effect of coloring beverages and the subsequent bleaching on CAD/CAM nanohybrid and nanoceramic restorative materials [17]. Karakaş and Küden compared the optical properties of cements and resins after exposure to colorant beverages [22]. Furthermore, external agents may also influence the translucency of monolithic esthetic materials [23].

The aim of this laboratory-based research is to determine and compare the color change of various new commercial esthetic monolithic monochromatic CAD/CAM materials using a spectrophotometer after thermocycling and exposure to staining solutions. A further aim is to evaluate the color changes as a function of the exposure time to the staining solutions. The null hypothesis of this study was that there is no difference in the color stability among the esthetic monolithic monochromatic CAD/CAM materials after thermocycling and exposure to staining solutions.

## 2. Materials and Methods

Various materials have been developed in dental technology that offer both durability and esthetics in dental prosthetics. Esthetic materials that are very similar to natural teeth in terms of color and translucency are becoming increasingly important. We therefore decided to investigate the optical properties of the latest hybrid materials and their durability. Five esthetic monolithic monochromatic restorative materials were selected for this in vitro study—CAD/CAM nanoceramic (GC Cerasmart270), CAD/CAM lithium disilicate ceramic (IPS e.max CAD), CAD/CAM polymer (Telio CAD), CAD/CAM composite (Tetric CAD) and CAD/CAM polymer-infiltrated ceramic (Vita Enamic). The technical profiles and manufacturers of the materials are listed in Table 1.

A total of 200 samples were prepared, with 40 samples per restorative material. Within each material group, 20 samples measuring 12 × 14 × 1 mm and 20 samples measuring 12 × 14 × 2 mm were prepared.

The samples were cut from each CAD/CAM block using a water-cooled, low-speed diamond saw (Isomet 1000, Buehler, Lake Bluff, IL, USA). The top and bottom surfaces of the samples were polished and wet ground with the sequential use of silicon carbide paper P180, P280, P800, P1500 and P4000 (Sia Abrasives, Frauenfeld, Switzerland) for three minutes. The top and bottom surfaces were then polished for 3 min with a 0.05 micron aluminum oxide suspension (MasterPrep; Buehler, Lake Bluff, IL, USA) and a polishing cloth (MasterTex; Buehler). Finally, all samples were rinsed under water and dried with compressed air. All IPS e.max CAD preparations were crystallized in a furnace (Programat EP3010, Ivoclar Vivadent AG, Schaan, Liechtenstein) according to the manufacturer’s instructions. 

### 2.1. Thermocycling and Staining Solutions

Artificial aging in distilled water of all 200 samples was carried out using a thermocycler (THE-1200; SD Mechatronik, Feldkirchen-Westerham, Germany) with 5000 thermocycles at 5 °C and 55 °C, a dwell time of 20 s and a dripping time of 5 s. After thermocycling in distilled water, all samples were immersed in staining solutions. 

The staining solutions were instant coffee (Nescafe Gold Crema, Nestle France, Noisiel, France), black tea (Franck Ltd., Zagreb, Croatia) and red wine (Karizma 2019, Winery Petrač, Krapinske Toplice, Croatia), with distilled water as the control liquid. The instant coffee and black tea were prepared with tap water according to the manufacturer’s instructions. The samples were immersed in Petri dishes, each filled with 50 mL of a different staining solution.

Within each material group (*n* = 40), 20 samples with a thickness of 1 mm and 20 samples with a thickness of 2 mm were divided into four subgroups (*n* = 5) in order to be able to immerse the samples in four different staining solutions. For this purpose, 4 Petri dishes were filled with black tea, 4 with instant coffee, 4 with red wine and 4 with distilled water. The Petri dishes were stored in an incubator (Memmert 100–800, Memmert GmbH + Co. KG, Schwabach, Germany) at 37 °C for four weeks. The solutions were renewed every second day to avoid possible contamination with bacteria and yeasts. 

### 2.2. Determination of Color 

The color of the samples was determined using a spectrophotometer (Spectro Shade, Handy Dental, MHT, Verona, Italy) within the CIELAB space, defined by the International Commission on Illumination (EN ISO/CIE 11664-4:2019) [24]. The CIELAB coordinates (L*c*H*) of the samples were measured under D_65_ illuminant light (6503 K) according to the standard EN ISO 11664-2:2011 [25]. All samples were measured against a black and a white background to simulate clinical conditions. The black background simulates restorations that are not surrounded by cavity walls, i.e., anterior restorations and the white background simulates restorations that are surrounded by tooth walls. After placing the sample on a white or black background, the spectrophotometer was positioned over the sample using the extension (housing), which encloses the entire sample, so that the entire surface of the sample is visible on the screen of the device. The distance of the spectrophotometer from the sample was 3.5 cm. Before each measurement, the spectrophotometer was calibrated according to the manufacturer’s recommendations. The spectrophotometer was calibrated before each measurement using a white and a green ceramic tile. The white and green ceramic tiles are located on the spectrophotometer’s docking station. The device provides information on the success of the calibration. Collage paper (INTERDRUK, Warsaw, Poland) was used as the white and black background. 

Color determination with a spectrophotometer was carried out at the beginning of the study, i.e., before thermocycling and exposure to staining solutions. The samples were then subjected to aging and were subsequently immersed in the beverages for 1 week, 2 weeks, 3 weeks and 4 weeks. All the above measurements were taken on a white and black background.

Based on the L*c*H* values obtained, the color change (ΔE*_00_) of each sample is calculated using the CIELAB formula (EN ISO 11664-6:2016) [26]:

ΔE_00_ = [(ΔL′/(K_L_ S_L_))^2^ + (ΔC′/(K_C_ S_C_))^2^ + (ΔH′/(K_H_ S_H_))^2^ + R_T_ (ΔC′/(K_C_ S_C_)) (ΔH′/(K_H_ S_H_))]^1/2^ (1)

ΔL′, ΔC′ and ΔH′ are the differences in lightness, chroma and hue between two compared specimens. S_L_, S_C_ and S_H_ are the weighing functions for the lightness, chroma and hue components. K_L_, K_C_ and K_H_ are the parametric factors that are adjusted according to the different viewing parameters. R_T_ is the rotation function that specifies the interaction between chroma and hue differences in the blue range.

The CIEDE 2000 perceptibility threshold (PT) used was ΔE_00_ = 0.8, while the acceptance threshold (AT) was ΔE_00_ = 1.8 [4,27].

### 2.3. Statistical Analysis

The sample size was calculated to compare the color change between 5 material types, 4 liquid types and 2 sample thicknesses (three-factorial ANOVA). The significance of the test was set at 0.8 and the significance level at 0.05. The sample size was chosen so that a medium effect could be demonstrated (Cohen’s f = 0.25). For the factor sample thickness, the required sample size is 129; for the factor material, the required total sample size is 180 and for the factor liquid, the required sample size is 197. As the number of groups is 40, the total sample size is 200 (5 per group). The sample size was calculated using G*Power 3.1.9.7. The results obtained were statistically analyzed. The mean value and standard deviation are used to describe the color change for different materials, staining solution, backgrounds, sample thickness and aging stages. The agreement of the data with the normal distribution was tested using the Shapiro–Wilk test with Bonferroni correction. A mixed factorial ANOVA model was used to test the differences in color change between different materials, staining solutions, backgrounds, sample thickness and aging stages. The color change at 5 different aging stages and for different backgrounds was measured on the same samples and are factors within the mixed ANOVA design. Post hoc comparisons were performed using Tukey’s test. 

Analyses were performed using the SAS statistical package on the Windows platform. All tests were performed with a significance level of α = 0.05.

## 3. Results

**Figure 1:**
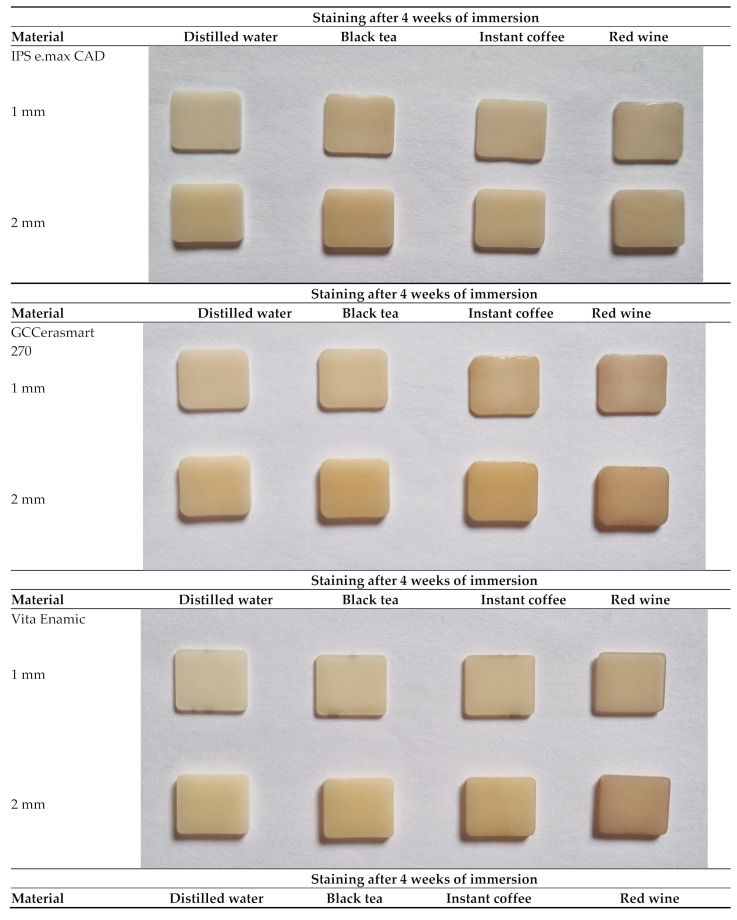

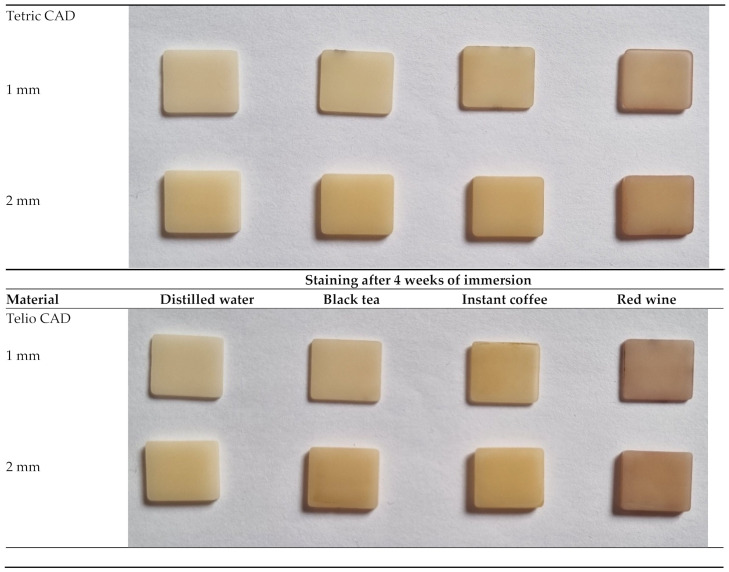
Color changes of all materials used in this study after 4 weeks of immersion in different staining solutions.

Figure 1 shows the color changes of all the materials used in this study after 4 weeks of immersion in different staining solutions.

**Figure 2:**
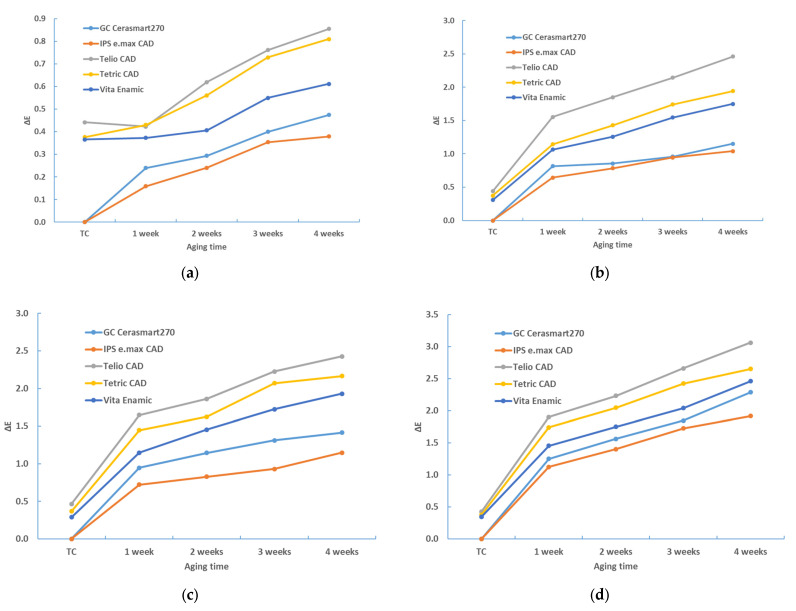
Descriptive statistics—color change (ΔE*_00_)—1 mm—white background: (**a**) distilled water; (**b**) black tea; (**c**) instant coffee; (**d**) red wine.

**Figure 3:**
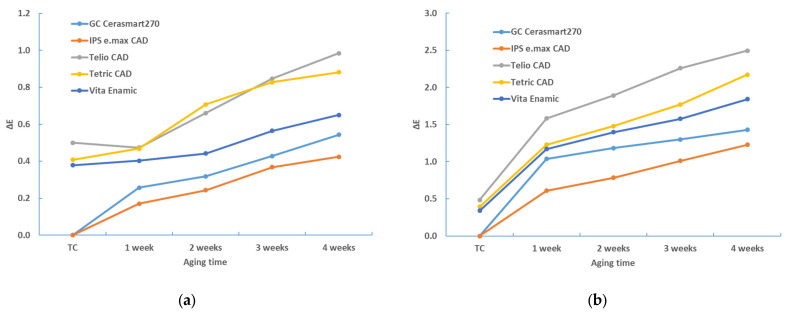

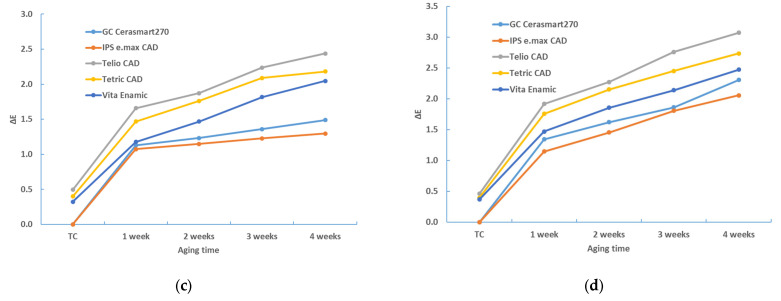
Descriptive statistics—color change (ΔE*_00_)—1 mm—black background: (**a**) distilled water; (**b**) black tea; (**c**) instant coffee; (**d**) red wine.

**Figure 4:**
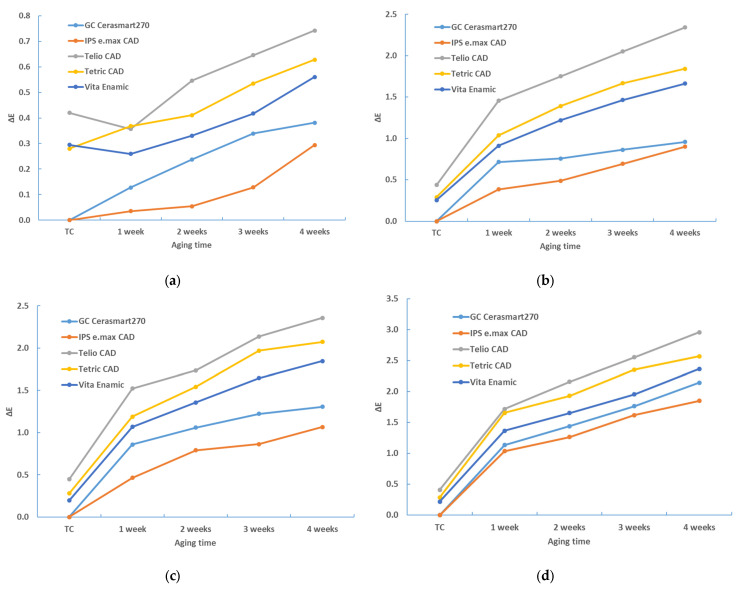
Descriptive statistics—color change (ΔE*_00_)—2 mm—white background: (**a**) distilled water; (**b**) black tea; (**c**) instant coffee; (**d**) red wine.

**Figure 5:**
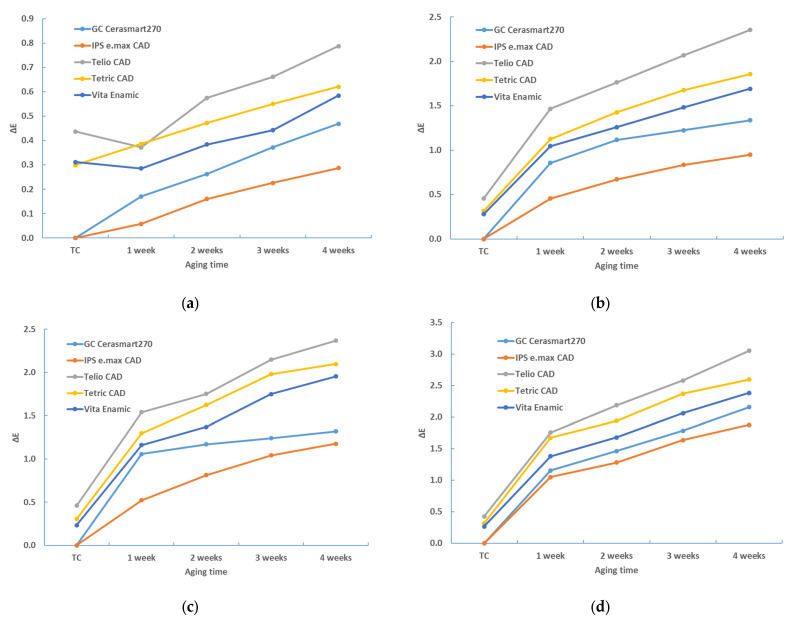
Descriptive statistics—color change (ΔE*_00_)—2 mm—black background: (**a**) distilled water; (**b**) black tea; (**c**) instant coffee; (**d**) red wine.

**Figure 6:**
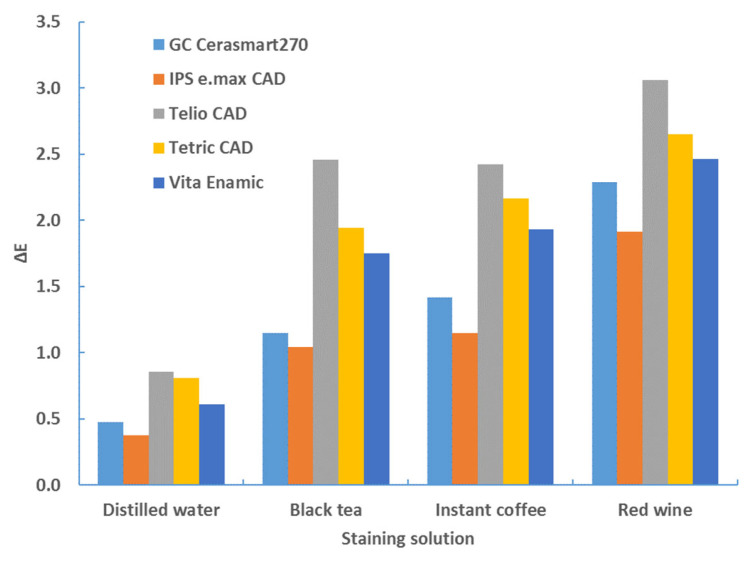
ANOVA multiple comparisons—color change (ΔE*_00_)—1 mm—white background—after 4 weeks.

**Figure 7:**
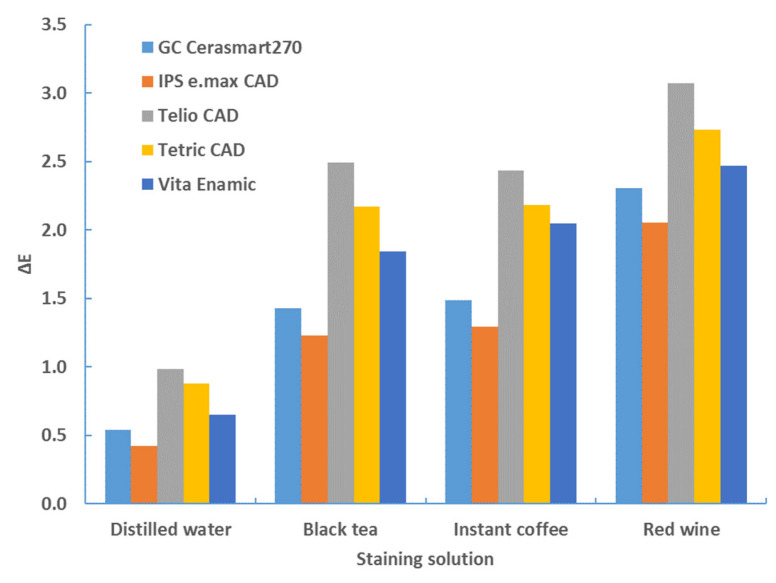
ANOVA multiple comparisons—color change (ΔE*_00_)—1 mm—black background—after 4 weeks.

Table 2, Table 3, Table 4, Table 5, Table 6 and Table 7 and Figure 2, Figure 3, Figure 4, Figure 5, Figure 6 and Figure 7 contain descriptive statistics (mean and standard deviation) for five materials and four staining solutions for values measured immediately after thermocycling (TC) and after 1, 2, 3 and 4 weeks. The results of the color change for the white and black background and for sample thicknesses of 1 mm and 2 mm are shown separately.

The influence of material type, staining solution, color background, sample thickness and aging time on the color change was tested using a mixed factorial ANOVA model.

The ANOVA test confirmed that all factors were significant. The type of material, the staining solution, the sample thickness, the color background and the aging time influenced the color change (Table 6, *p* < 0.0001). Of all the interactions between material type, type of staining solution, color background and sample thickness, only the interaction between material type and type of staining solution (material*staining solution, Table 6) is significant, i.e., the extent of the color change depends on the combination of material type and of the staining solution.

The combined influence of material type and staining solution on the color change was tested using Tukey’s test (for multiple comparisons). The color change between all material types for each staining solution and between all staining solutions for each material type was compared using the data measured after 4 weeks. There is a significant difference between all material types and between all staining solutions. The only exception is Telio CAD, where no difference was found between black tea and coffee.

**Figure 8:**
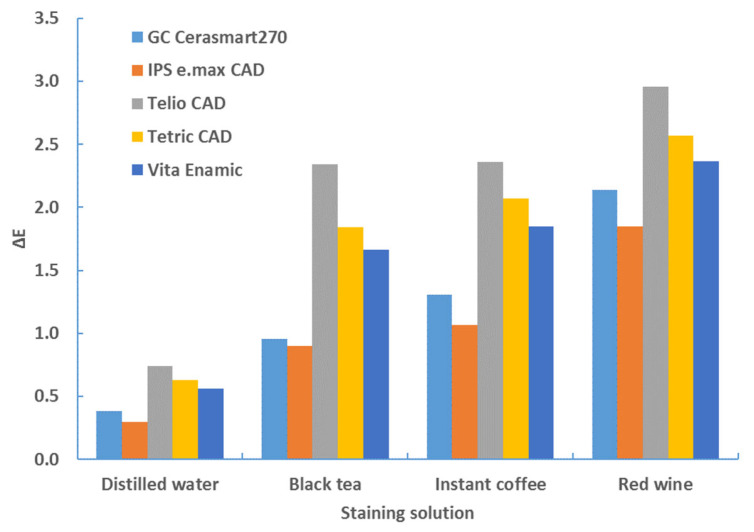
ANOVA multiple comparisons—color change (ΔE*_00_)—2 mm—white background—after 4 weeks.

**Figure 9:**
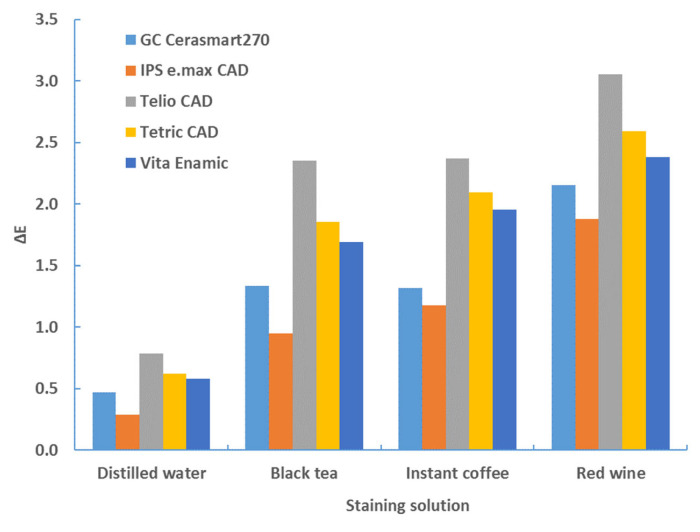
ANOVA multiple comparisons—color change (ΔE*_00_)—2 mm—black background—after 4 weeks.

Table 7, Table 8, Table 9 and Table 10 and Figure 6, Figure 7, Figure 8 and Figure 9 provide a detailed comparison of the color change between material type and staining solution.

Table 7 clearly shows that the color change with a sample thickness of 1 mm and a white background is the greatest with Telio CAD, followed by Tetric CAD, Vita Enamic, GC Cerasmart270 and IPS e.max CAD. The comparison of the staining solutions shows that the greatest color change occurs with red wine, followed by coffee and black tea for all materials. The least color change was observed with distilled water.

The comparison of the color change for the black background leads to the same result as the comparison for the white background (Table 8). The order for both, the type of material and the staining solution is unchanged (Table 8, Figure 6).

The same comparisons were made with the 2 mm thick samples. The results are the same; the order of material types and staining solutions remains unchanged (Table 9 and Figure 7 for white background and Table 10 and Figure 8 for black background).

The comparison of the effects of the material type on the color change clearly shows the following sequence: Telio CAD > Tetric CAD > Vita Enamic > GC Cerasmart 270 > IPS e.max CAD, while the comparison of the influence of the staining solutions on the color change shows that red wine > instant coffee ≥ black tea > distilled water.

The color change for instant coffee and black tea does not differ for the Telio CAD material.

**Figure 10:**
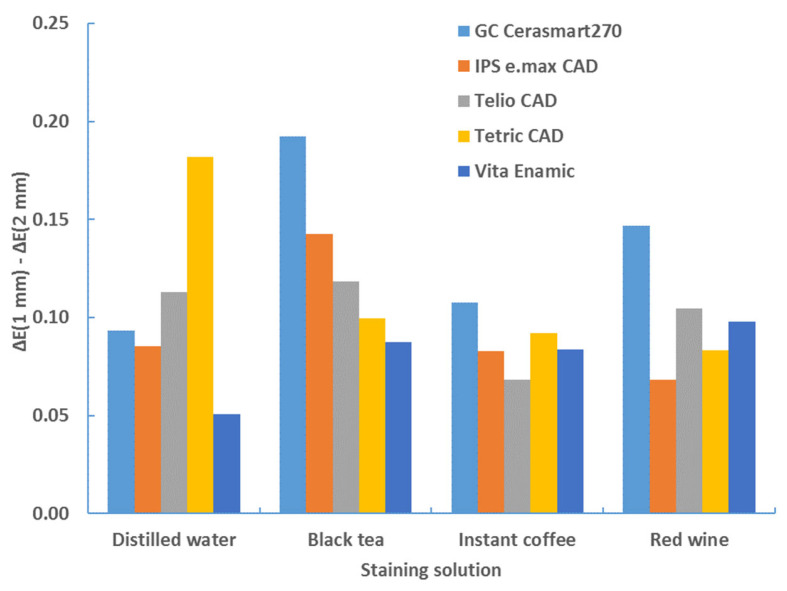
Difference in color change (ΔE*_00_) between samples of 1 mm and 2 mm thickness—white background—after 4 weeks.

**Figure 11:**
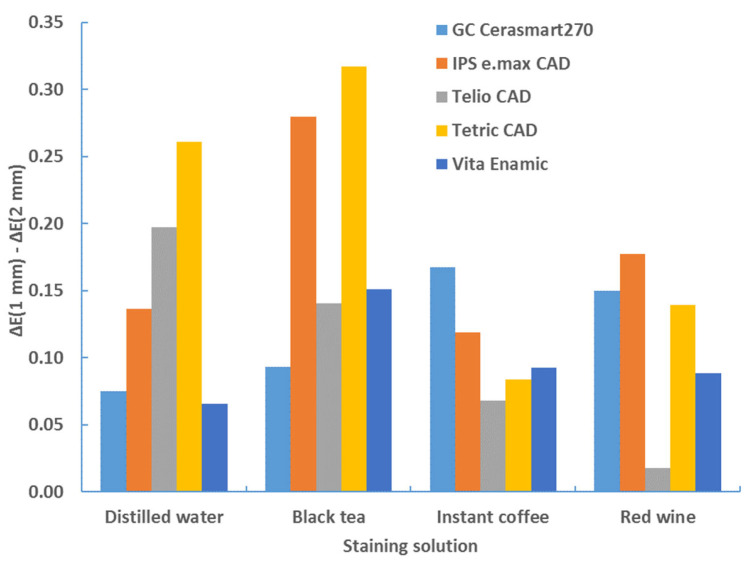
Difference in color change (ΔE*_00_) between samples of 1 mm and 2 mm thickness—black background—after 4 weeks.

The sample thickness has a significant effect on the color change for each type of material, staining solution and background (*p* < 0.0001, ANOVA test; Table 6). Table 7, Table 8, Table 9 and Table 10 show that the color change is greater for the samples with a thickness of 1 mm. This comparison is clearly illustrated in Table 11 and Table 12 and Figure 10 and Figure 11. The difference in color change between samples with a thickness of 1 mm and samples with a thickness of 2 mm was shown, as follows: ΔE*_00_ (1 mm)–ΔE*_00_ (2 mm).

The difference is positive for all combinations of material types, staining solutions and background colors.

**Figure 12:**
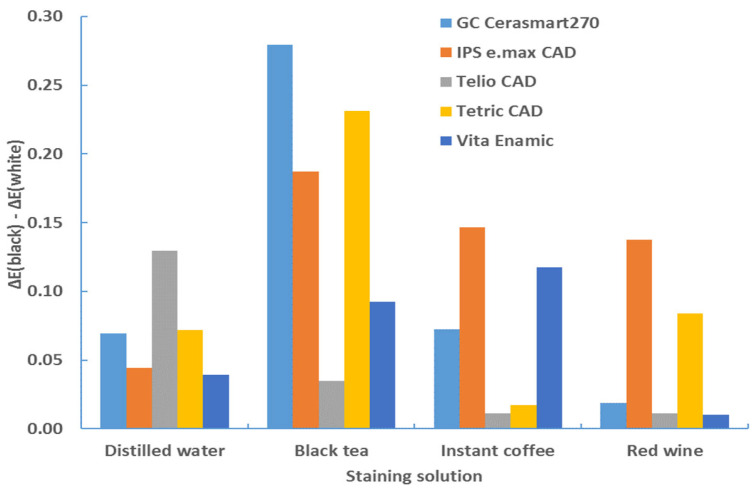
Difference in color change (ΔE*_00_) between black and white background—1 mm—after 4 weeks.

**Figure 13:**
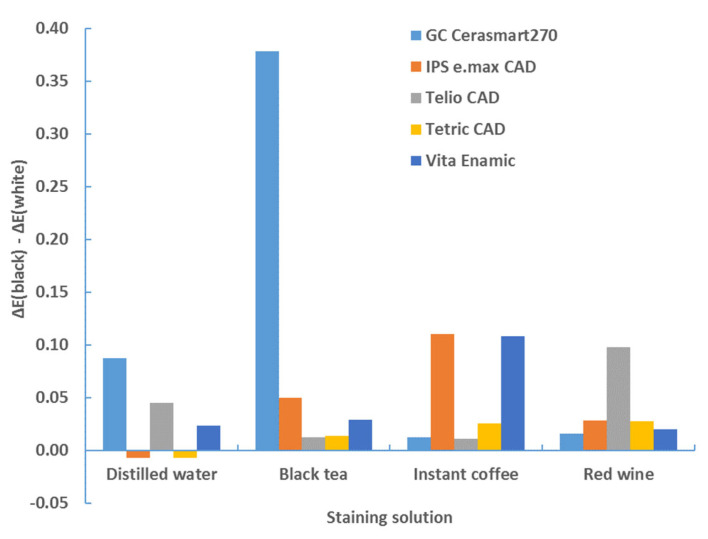
Difference in color change (ΔE*_00_) between black and white background—2 mm—after 4 weeks.

Similarly, a difference in color change for the white and black background was observed. The color change between two backgrounds is significantly different (*p* < 0.0001, ANOVA test; Table 6) and was taken into account. Table 7, Table 8, Table 9 and Table 10 show that the color change is greater with a black background. This comparison is clearly illustrated in Table 13 and Table 14 and Figure 12 and Figure 13. The difference in color change between the results on a black background and the results on a white background—expressed as ΔE*_00_ (black)–ΔE*_00_ (white)—was shown.

The difference is positive for all combinations of material types, staining solutions and sample thickness, except for distilled water on the 2 mm wide samples and for the materials IPS e.max. CAD and TETRIC CAD, where the color change was greater on a white background (for 0.01).

The aging time also has a significant influence on the color change (*p* < 0.0001, ANOVA test; Table 6). Table 2, Table 3, Table 4 and Table 5, and Figure 1, Figure 2, Figure 3 and Figure 4 show that the color change increases with time.

## 4. Discussion

From the results presented, it is clear that red wine causes the strongest discoloration. However, the aim of this study is to determine the degree of discoloration, in particular whether the color change is clinically acceptable. Therefore, the color stability and durability of the latest CAD/CAM materials were investigated in this study. 

Due to their natural and esthetic appearance, low thermal conductivity, good hygienic properties and wear resistance, dental ceramics are among the most popular restorative materials in prosthetics. Composites are another widely used esthetic restorative material in dentistry. Compared to ceramics, composites are less brittle but have a lower color stability and a higher wear, which limits their use. The disadvantages of these popular dental restorative materials have led to the development of new biomaterials that mimic the physical properties of natural teeth. Polymer-infiltrated ceramic network (PICN) materials, also known as hybrid ceramics, have been developed to compensate for the disadvantages of ceramics and composites. PICN materials are expected to be less brittle, have a higher flexural strength and stiffness, a high strength and are easy to mill with CAD/CAM systems. In addition, their physical properties are similar to those of natural teeth and cause less abrasion on the opposing teeth compared to dental ceramics. The long-term goal of PICN materials is to fully replicate the physical properties of natural teeth. In line with this goal, new PICN materials are available on the dental market. Knowledge about the optical properties of PICN is limited and there are few sources in the specialized literature. In addition, CAD/CAM blocks offer a higher quality due to the standardized industrial production process [8,28].

This laboratory-based study investigated the effects of artificial aging, staining solutions and exposure time on the color stability of five different monolithic monochromatic CAD/CAM materials, including CAD/CAM hybrid ceramic, CAD/CAM composite, CAD/CAM lithium disilicate ceramic and CAD/CAM polymer, and revealed statistically significant differences. The null hypothesis was refuted in this study, as exposure to different staining solutions showed a remarkable effect on the color change of certain CAD/CAM materials, which was significantly dependent on the type of staining solution, the materials used and the immersion time. In addition to the importance of matching, the long-term stability of the color must also be taken into consideration when selecting a restorative material, especially in the case of newly developed dental materials [7]. The coloring agents were black tea, instant coffee and red wine, while distilled water was used as a control agent. The thickness of the samples was either 1 mm or 2 mm, and the color shade of all selected CAD/CAM materials was A2/2M2. The color measurements of each sample were performed on a white and black background under a D_65_ light source (6503 K). The color changes (ΔE*_00_) of each sample were calculated based on the obtained L*c*H* values. The results showed that the color change (∆E*) is below 3.7 for all materials. In most studies, it is accepted that differences in spectrophotometer color change values (∆E*) of less than 3.7 are considered clinically acceptable [9]. Since all color change values in this study were below 3.7 for all materials tested, they are considered satisfactory. This means that the color of the tested materials is stable under all experimental conditions used in this study. The color stability of all tested materials can be attributed to the fact that CAD/CAM materials can be polished better, do not have a porous structure and absorb less water than PMMA resins, for example.

Vita Enamic and GC Cerasmart 270 are categorized as a resin-based material, whose matrix consists of a polymer-infiltrated ceramic network. This ceramic core and its resistance to water absorption probably explain the lack of discoloration observed in these materials. In contrast, Telio CAD has a PMMA-based matrix, which is relatively hydrophilic and promotes a higher level of water absorption. The susceptibility of CAD/CAM composites such as Tetric CAD to discoloration may be influenced by factors such as the extent of water absorption and the hydrophilicity of the composite matrix [29]. Overall, CAD/CAM materials based on composite or hybrid materials exhibited lower color stability than glass–ceramic materials. It is generally assumed that glass–ceramic CAD/CAM materials have a higher color stability than composite-based CAD/CAM materials. This is consistent with our results, as IPS e.max CAD shows better color stability compared to composite-based CAD/CAM materials. The color stability of glass–ceramic materials can be influenced by various factors, including the crystalline structure, grain size and porosity [30,31].

Our study confirms earlier research findings that red wine causes the strongest discoloration of CAD/CAM materials. Seyidaliyeva et al. compared the color stability of polymer-infiltrated ceramics with lithium disilicate ceramics and composites. The strongest discoloration was observed in the samples immersed in red wine. The discoloration of hybrid ceramics was found to be intermediate between lithium disilicates and composites. In addition, thermocycling had an effect on the color change, which was, however, below human perceptibility. The conclusion of Seyidaliyeva’s study was that all factors investigated, such as material, staining solution and thermocycling, influence the discoloration [27,32].

Quek et al. compared the color changes of direct composite, indirect composite, CAD/CAM composite and hybrid ceramic materials. Direct, indirect and CAD/CAM composites can discolor to varying degrees after contact with coloring beverages. CAD/CAM composites appeared to behave better in red wine compared to direct and indirect composites, although the changes were still clinically perceptible [8].

Lauvahutanon et al. investigated the color difference of various CAD/CAM materials after immersion in coffee. The ΔE values after immersion in water did not change significantly, while the ΔE values after immersion in coffee increased significantly with increasing immersion time. The ΔE values of the material types after immersion in coffee were significantly different. The highest ΔE values were measured for a CAD/CAM polymer, followed by a hybrid ceramic block [33].

Peñate et al. determined similar ΔE values for a CAD/CAM polymer as the current study. The CAD/CAM polymer was immersed in red wine, instant coffee and black tea for 30 days. The discoloration was significant with red wine, followed by instant coffee and black tea [34].

The study by Kul et al., in which a CAD/CAM polymer was immersed in tea and coffee, also showed high ΔE values after 21 days in coloring agents [35].

Stamenkovic et al. [36] evaluate the effects of staining and aging on the color changes of CAD/CAM nanoceramics, CAD/CAM polymer-infiltrated ceramic networks (PICNs) and CAD/CAM lithium disilicate ceramics. The results of this study suggest that lithium disilicate ceramics were more color stable than resin nanoceramics and PICN materials when stained with coffee and red wine. In addition, the results of color change after accelerated aging are similar to the current study.

The staining caused by coffee and wine can be explained by the absorption of their pigments into the organic components of the materials [37]. Red wine contains alcohol, acids, chromogens and tannins, while tea is rich in tannins, and coffee is high in chromogens. Color changes are likely to be caused by a mixture of factors, including matrix degradation by alcohol and acids, infiltration and absorption of colorants into the material and surface adhesion of colorants due to increased surface roughness after immersion in coloring agents. Tea consists of oxalic acid, malic acid and citric acid, while coffee contains approximately 22 different acids. Of these, citric acid, acetic acid, malic acid and other high-molecular-weight acids contribute the most to the total acid content of coffee [8]. Studies have shown that popular drinks such as coffee, tea and red wine can significantly alter the microstructure of materials [38,39,40,41,42].

After immersion in red wine, instant coffee and black tea, the samples showed an increasing darkening of color with prolonged immersion. It is clear from all this that the chemical composition of the dental material has the greatest influence on color stability in different staining solutions. Glass–ceramics are more resistant to coloring agents than resin ceramics [43]. The results of this study show that the ceramic material IPS e.max CAD exhibits the highest color stability, and the polymer Telio CAD exhibits the lowest. The results were reported using the CIEDE2000 color difference formula (ΔE_00_), which has a better correlation with human color perception compared to the CIELab formula [44]. In this study, ΔE_00_ interpretations were made in terms of perceptible and acceptable thresholds. Accordingly, the CIEDE2000 threshold of perception (PT) was 0.8 and the threshold of acceptance (AT) was 1.8. These values have been included in ISO/TR 28642:201623 and should be applied to all issues related to the quality of tooth shade-matching in dentistry. These thresholds can serve as a measure for quality assessment and can help in the selection of aesthetic dental materials, in the evaluation of their performance in the clinical setting and in the interpretation of visual and instrumental observations both in dental research and in subsequent standardization processes [26,45].

The main limitation of this laboratory-based study of staining and accelerated artificial aging is that the oral environment cannot be fully replicated. Consequently, the results may not accurately reflect clinical variability, as the color stability of restorations in the mouth can be influenced by various factors such as oral hygiene, different beverage consumption and habits such as smoking. In addition, the underlying tooth structures can significantly influence the esthetic outcome of using these restorative materials. Even in clinical use, only the outer surface is exposed to external influences, while the inner surface is bonded to the underlying structure. However, the study represents a thorough investigation of the factors influencing the color change of dental materials and provides valuable insights for both researchers and practitioners in this field. Therefore, a limitation of this study is that the clinical environment and its effects on the discoloration of the materials used in this study were not fully simulated. Indeed, the color change was determined on both sides of the samples, in contrast to the clinical situation, where one side is attached to the tooth surface, resulting in a more intense discoloration. Considering the importance of color and optical properties in dental medicine, future studies should investigate these properties as a function of the microstructure and chemical composition of the material, using multilayer structures on different substrates. In addition, future studies should investigate the effects of staining solutions on the microstructure and mechanical properties of the materials studied.

## 5. Conclusions

Within the limits of this in vitro study, the conclusion is that color change is influenced by the type of material, the staining solution, the sample thickness, the color background and the aging time.

## Figures and Tables

**Table 1:** Technical profile and manufacturers of the restorative materials.

Material (Manufacturer)	LOT	Type	Composition
GC Cerasmart270 (GC Europe N.V., Leuven, Belgium)	210906	Hybrid ceramic (Nanoceramic/nanoparticles infiltrated in a polymer network)	71% silica and barium glass nanoparticles (Bis—MEPP ^6^, UDMA ^4^, DMA ^7^)
IPS e.max CAD (Ivoclar Vivadent, Schaan, Liechtenstein)	Z03B1G	Lithium disilicate-reinforced glass ceramic	80% SiO_2_ ^1^ 20% Li_2_O ^2^
Telio CAD (Ivoclar Vivadent, Schaan, Liechtenstein)	Z03X2L	CAD/CAM polymer	99.5% PMMA ^8^ <1.0 pigments
Tetric CAD (Ivoclar Vivadent, Schaan, Liechtenstein)	Z03R81	Nanohybrid composite	64% barium glass 7.1% SiO_2_ ^1^ 28.4% DMA ^7^ 0.5% pigments
Vita Enamic (Vita Zahnfabrik, Bad Säckingen, Germany)	95860	Hybrid ceramic (Polymer-infiltrated ceramic network)	86% ceramic (50–63% SiO_2_ ^1^; 20–23% Al_2_O_3_ ^3^) 14% polymer (UDMA ^4^ + TEGDMA ^5^)

^1^ SiO_2_: silicon dioxide; ^2^ Li_2_O: lithium oxide; ^3^ Al_2_O_3_: aluminum oxide; ^4^ UDMA: urethane dimethacrylate; ^5^ TEGDMA: triethylene glycol dimethacrylate; ^6^ Bis-MEPP: 2,2-Bis-(4-methacryloyl-ethoxyphenyl) propane; ^7^ DMA: dimethacrylate; ^8^ PMMA: polymethyl methacrylate (PMMA).

**Table 2:** Descriptive statistics—color change (ΔE*_00_)—1 mm—white background.

Material	TC In Distilled Water	Staining Solution	1 Week	2 Weeks	3 Weeks	4 Weeks
	Mean St.dev.		Mean St.dev.	Mean St.dev.	Mean St.dev.	Mean St.dev.
GC Cerasmart270	0.00 0.00	Distilled water	0.24 0.03	0.29 0.01	0.40 0.01	0.47 0.02
	0.00 0.00	Black tea	0.82 0.07	0.86 0.07	0.96 0.03	1.15 0.04
	0.00 0.00	Instant coffee	0.95 0.05	1.15 0.13	1.31 0.07	1.41 0.07
	0.00 0.00	Red wine	1.25 0.03	1.56 0.01	1.84 0.04	2.29 0.02
IPS e.max CAD	0.00 0.00	Distilled water	0.16 0.03	0.24 0.03	0.35 0.03	0.38 0.06
	0.00 0.00	Black tea	0.64 0.04	0.78 0.00	0.94 0.04	1.04 0.03
	0.00 0.00	Instant coffee	0.72 0.03	0.83 0.02	0.93 0.04	1.15 0.01
	0.00 0.00	Red wine	1.13 0.01	1.40 0.04	1.72 0.07	1.92 0.07
Telio CAD	0.44 0.03	Distilled water	0.43 0.02	0.62 0.01	0.76 0.03	0.86 0.06
	0.44 0.02	Black tea	1.14 0.04	1.85 0.03	2.14 0.03	2.46 0.04
	0.46 0.03	Instant coffee	1.45 0.04	1.86 0.04	2.23 0.04	2.43 0.03
	0.43 0.02	Red wine	1.74 0.03	2.23 0.05	2.66 0.03	3.06 0.03
Tetric CAD	0.38 0.03	Distilled water	0.43 0.02	0.56 0.02	0.73 0.04	0.81 0.06
	0.37 0.03	Black tea	1.14 0.04	1.43 0.01	1.74 0.03	1.94 0.03
	0.37 0.03	Instant coffee	1.45 0.04	1.62 0.02	2.07 0.01	2.16 0.03
	0.37 0.05	Red wine	1.74 0.03	2.05 0.03	2.42 0.01	2.65 0.03
Vita Enamic	0.37 0.01	Distilled water	0.37 0.01	0.41 0.01	0.55 0.02	0.61 0.02
	0.31 0.04	Black tea	1.07 0.02	1.26 0.02	1.55 0.03	1.75 0.02
	0.29 0.07	Instant coffee	1.15 0.03	1.45 0.03	1.72 0.02	1.93 0.03
	0.34 0.03	Red wine	1.46 0.02	1.75 0.03	2.04 0.03	2.46 0.03

**Table 3:** Descriptive statistics—color change (ΔE*_00_)—1 mm—black background.

Material	TC In Distilled Water	Staining Solution	1 Week	2 Weeks	3 Weeks	4 Weeks
	Mean St.dev.		Mean St.dev.	Mean St.dev.	Mean St.dev.	Mean St.dev.
GC Cerasmart270	0.00 0.00	Distilled water	0.26 0.04	0.32 0.01	0.43 0.02	0.54 0.02
	0.00 0.00	Black tea	1.04 0.14	1.17 0.07	1.30 0.04	1.43 0.03
	0.00 0.00	Instant coffee	1.13 0.03	1.23 0.08	1.36 0.06	1.49 0.06
	0.00 0.00	Red wine	1.34 0.03	1.62 0.03	1.86 0.04	2.31 0.02
IPS e.max CAD	0.00 0.00	Distilled water	0.17 0.02	0.24 0.04	0.37 0.08	0.42 0.02
	0.00 0.00	Black tea	0.61 0.09	0.78 0.02	1.01 0.05	1.23 0.06
	0.00 0.00	Instant coffee	1.07 0.03	1.15 0.08	1.23 0.09	1.30 0.11
	0.00 0.00	Red wine	1.14 0.01	1.45 0.01	1.81 0.05	2.05 0.04
Telio CAD	0.50 0.03	Distilled water	0.47 0.02	0.66 0.03	0.85 0.02	0.98 0.01
	0.49 0.01	Black tea	1.58 0.02	1.89 0.02	2.26 0.02	2.49 0.01
	0.49 0.03	Instant coffee	1.66 0.02	1.87 0.04	2.24 0.04	2.44 0.03
	0.46 0.02	Red wine	1.92 0.02	2.27 0.03	2.76 0.04	3.07 0.03
Tetric CAD	0.41 0.02	Distilled water	0.47 0.02	0.71 0.01	0.83 0.01	0.88 0.01
	0.39 0.02	Black tea	1.23 0.01	1.48 0.02	1.77 0.01	2.17 0.03
	0.40 0.01	Instant coffee	1.47 0.03	1.76 0.02	2.09 0.01	2.18 0.02
	0.40 0.04	Red wine	1.76 0.03	2.15 0.02	2.45 0.01	2.73 0.04
Vita Enamic	0.38 0.02	Distilled water	0.40 0.01	0.44 0.02	0.56 0.03	0.65 0.03
	0.34 0.05	Black tea	1.17 0.02	1.40 0.03	1.58 0.02	1.84 0.04
	0.32 0.05	Instant coffee	1.18 0.02	1.47 0.02	1.82 0.01	2.05 0.04
	0.37 0.03	Red wine	1.47 0.02	1.85 0.03	2.14 0.01	2.47 0.03

**Table 4:** Descriptive statistics—color change (ΔE*_00_)—2 mm—white background.

Material	TC In Distilled Water	Staining Solution	1 Week	2 Weeks	3 Weeks	4 Weeks
	Mean St.dev.		Mean St.dev.	Mean St.dev.	Mean St.dev.	Mean St.dev.
GC Cerasmart270	0.00 0.00	Distilled water	0.13 0.03	0.24 0.04	0.34 0.02	0.38 0.02
	0.00 0.00	Black tea	0.72 0.07	0.76 0.07	0.86 0.03	0.96 0.03
	0.00 0.00	Instant coffee	0.86 0.04	1.06 0.15	1.22 0.09	1.31 0.10
	0.00 0.00	Red wine	1.14 0.03	1.44 0.02	1.76 0.03	2.14 0.04
IPS e.max CAD	0.00 0.00	Distilled water	0.04 0.01	0.05 0.01	0.13 0.02	0.29 0.01
	0.00 0.00	Black tea	0.39 0.01	0.49 0.11	0.69 0.09	0.90 0.06
	0.00 0.00	Instant coffee	0.46 0.05	0.79 0.02	0.86 0.02	1.07 0.02
	0.00 0.00	Red wine	1.03 0.02	1.26 0.03	1.62 0.01	1.85 0.02
Telio CAD	0.42 0.02	Distilled water	0.36 0.03	0.55 0.02	0.65 0.03	0.74 0.03
	0.44 0.01	Black tea	1.45 0.03	1.75 0.03	2.05 0.02	2.34 0.04
	0.45 0.04	Instant coffee	1.52 0.02	1.74 0.03	2.14 0.02	2.36 0.04
	0.41 0.01	Red wine	1.72 0.02	2.16 0.03	2.55 0.03	2.96 0.03
Tetric CAD	0.28 0.05	Distilled water	0.37 0.01	0.41 0.01	0.53 0.03	0.63 0.01
	0.29 0.02	Black tea	1.04 0.01	1.39 0.01	1.67 0.03	1.84 0.03
	0.28 0.07	Instant coffee	1.19 0.02	1.54 0.03	1.97 0.02	2.07 0.04
	0.29 0.05	Red wine	1.65 0.03	1.93 0.03	2.35 0.04	2.57 0.01
Vita Enamic	0.29 0.02	Distilled water	0.26 0.03	0.33 0.03	0.42 0.03	0.56 0.02
	0.26 0.04	Black tea	0.91 0.06	1.22 0.01	1.46 0.03	1.66 0.03
	0.20 0.02	Instant coffee	1.07 0.03	1.36 0.02	1.65 0.04	1.85 0.03
	0.22 0.03	Red wine	1.37 0.03	1.65 0.01	1.96 0.05	2.36 0.03

**Table 5:** Descriptive statistics—color change (ΔE*_00_)—2 mm—black background.

Material	TC In Distilled Water	Staining Solution	1 Week	2 Weeks	3 Weeks	4 Weeks
	Mean St.dev.		Mean St.dev.	Mean St.dev.	Mean St.dev.	Mean St.dev.
GC Cerasmart270	0.00 0.00	Distilled water	0.17 0.02	0.26 0.04	0.37 0.01	0.47 0.02
	0.00 0.00	Black tea	0.86 0.01	1.11 0.06	1.22 0.05	1.34 0.03
	0.00 0.00	Instant coffee	1.06 0.01	1.17 0.05	1.24 0.10	1.32 0.10
	0.00 0.00	Red wine	1.16 0.03	1.46 0.02	1.78 0.02	2.16 0.03
IPS e.max CAD	0.00 0.00	Distilled water	0.06 0.02	0.16 0.03	0.23 0.07	0.29 0.08
	0.00 0.00	Black tea	0.46 0.07	0.67 0.04	0.83 0.04	0.95 0.04
	0.00 0.00	Instant coffee	0.52 0.04	0.81 0.03	1.04 0.02	1.18 0.02
	0.00 0.00	Red wine	1.05 0.01	1.28 0.02	1.64 0.01	1.88 0.02
Telio CAD	0.44 0.04	Distilled water	0.37 0.02	0.58 0.02	0.66 0.03	0.79 0.01
	0.45 0.02	Black tea	1.47 0.03	1.76 0.03	2.07 0.02	2.35 0.04
	0.46 0.03	Instant coffee	1.54 0.01	1.75 0.03	2.15 0.01	2.37 0.03
	0.43 0.01	Red wine	1.76 0.03	2.19 0.01	2.58 0.04	3.05 0.02
Tetric CAD	0.30 0.05	Distilled water	0.39 0.01	0.47 0.03	0.55 0.04	0.62 0.05
	0.31 0.01	Black tea	1.13 0.02	1.43 0.01	1.68 0.03	1.86 0.03
	0.31 0.06	Instant coffee	1.30 0.08	1.62 0.01	1.98 0.02	2.10 0.04
	0.32 0.06	Red wine	1.67 0.03	1.94 0.03	2.37 0.04	2.59 0.01
Vita Enamic	0.31 0.02	Distilled water	0.29 0.02	0.38 0.01	0.44 0.03	0.58 0.01
	0.28 0.04	Black tea	1.04 0.02	1.26 0.03	1.48 0.02	1.69 0.01
	0.23 0.04	Instant coffee	1.16 0.03	1.37 0.02	1.75 0.03	1.95 0.03
	0.27 0.03	Red wine	1.38 0.03	1.68 0.02	2.06 0.03	2.38 0.03

**Table 6:** ANOVA table—color change (ΔE*_00_).

Source	Degrees of Freedom	Sum of Squares	Mean Sum of Squares	F	*p*-Value
Model	83	1047.25	12.62	183.74	<0.0001
Material	4	168.84	42.21	614.68	<0.0001
Staining solution	3	377.46	125.82	1832.24	<0.0001
Material*Staining solution	12	13.73	1.14	16.66	<0.0001
Sample thickness	1	5.64	5.64	82.19	<0.0001
Material*Sample thickness	4	0.20	0.05	0.72	0.57
Staining solution*Sample thickness	3	0.01	0.00	0.04	0.99
Material*Staining solution*Sample thickness	12	0.18	0.02	022	1.00
Background color	1	1.76	1.76	25.66	<0.0001
Material*Background color	4	0.21	0,05	0.76	0.55
Staining solution*Background color	3	0.27	0.09	1.30	0.27
Sample thickness*Background color	1	0.04	0.04	0.58	0.45
Material*Staining solution*Background color	12	0.75	0.06	0.92	0.53
Material*Sample thickness*Background color	4	0.02	0.00	0.07	0.99
Staining solution*Sample thickness*Background color	3	0.01	0.00	0.02	0.99
Material*Staining solution*Sample thickness*Background color	12	0.18	0.02	0.22	1.00
Aging time	4	477.95	119.49	1740.03	<0.0001
Error	1916	131.57	0.07		
Total	1999	1178.82			

**Table 7:** ANOVA multiple comparison—color change (ΔE*_00_)—1 mm—white background—after 4 weeks.

Material	ΔE*_00_ in Various Staining Solutions			
	ΔE*_00_ in Distilled Water	ΔE*_00_ in Black Tea	ΔE*_00_ in Instant Coffee	ΔE*_00_ in Red Wine
GC Cerasmart270	0.47	1.15	1.41	2.29
IPS e.max CAD	0.38	1.04	1.15	1.92
Telio CAD	0.86	2.46 ^a^	2.43 ^a^	3.06
Tetric CAD	0.81	1.94	2.16	2.65
Vita Enamic	0.61	1.75	1.93	2.46

^a^ Tukey’s post hoc test; the same letter designates staining solutions with the same color change.

**Table 8:** ANOVA multiple comparison—color change (ΔE*_00_)—1 mm—black background—after 4 weeks.

Material	ΔE*_00_ in Various Staining Solution			
	ΔE*_00_ in Distilled Water	ΔE*_00_ in Black Tea	ΔE*_00_ in Instant Coffee	ΔE*_00_ in Red Wine
GC Cerasmart270	0.54	1.43	1.49	2.31
IPS e.max CAD	0.42	1.23	1.30	2.05
Telio CAD	0.98	2.49 ^a^	2.44 ^a^	3.07
Tetric CAD	0.88	2.17	2.18	2.73
Vita Enamic	0.65	1.84	2.05	2.47

^a^ Tukey’s post hoc test; the same letter designates staining solutions with the same color change.

**Table 9:** ANOVA multiple comparison—color change (ΔE*_00_)—2 mm—white background—after 4 weeks.

Material	ΔE*_00_ in Various Staining Solution			
	ΔE*_00_ in Distilled Water	ΔE*_00_ in Black Tea	ΔE*_00_ in Instant Coffee	ΔE*_00_ In Red Wine
GC Cerasmart270	0.38	0.96	1.31	2.14
IPS e.max CAD	0.29	0.90	1.07	1.85
Telio CAD	0.74	2.34 ^a^	2.36 ^a^	2.96
Tetric CAD	0.63	1.84	2.07	2.57
Vita Enamic	0.56	1.66	1.85	2.36

^a^ Tukey’s post hoc test; the same letter designates staining solutions with the same color change.

**Table 10:** ANOVA multiple comparison—color change (ΔE*_00_)—2 mm—black background—after 4 weeks.

Material	ΔE*_00_ in Various Staining Solution			
	ΔE*_00_ in Distilled Water	ΔE*_00_ in Black Tea	ΔE*_00_ in Instant Coffee	ΔE*_00_ in Red Wine
GC Cerasmart270	0.47	1.34	1.32	2.16
IPS e.max CAD	0.29	0.95	1.18	1.88
Telio CAD	0.79	2.35 ^a^	2.37 ^a^	3.05
Tetric CAD	0.62	1.86	2.10	2.59
Vita Enamic	0.58	1.69	1.95	2.38

^a^ Tukey’s post hoc test; the same letter designates staining solutions with the same color change.

**Table 11:** Difference in color change (ΔE*_00_) between samples of 1 mm and 2 mm thickness—white background—after 4 weeks.

Material	ΔE*_00_ in Various Staining Solution			
	ΔE*_00_ in Distilled Water	ΔE*_00_ in Black Tea	ΔE*_00_ in Instant Coffee	ΔE*_00_ in Red Wine
GC Cerasmart270	0.09	0.19	0.11	0.15
IPS e.max CAD	0.09	0.14	0.08	0.07
Telio CAD	0.11	0.12	0.07	0.10
Tetric CAD	0.18	0.10	0.09	0.08
Vita Enamic	0.05	0.09	0.08	0.10

**Table 12:** Difference in color change (ΔE*_00_) between samples of 1 mm and 2 mm thickness—black background—after 4 weeks.

Material	ΔE*_00_ in Various Staining Solution			
	ΔE*_00_ in Distilled Water	ΔE*_00_ in Black Tea	ΔE*_00_ in Instant Coffee	ΔE*_00_ in Red Wine
GC Cerasmart270	0.07	0.09	0.17	0.15
IPS e.max CAD	0.14	0.28	0.12	0.18
Telio CAD	0.20	0.14	0.07	0.02
Tetric CAD	0.26	0.32	0.08	0.14
Vita Enamic	0.07	0.15	0.09	0.09

**Table 13:** Difference in color change (ΔE*_00_) between black and white background—1 mm—after 4 weeks.

Material	ΔE*_00_ in Various Staining Solution			
	ΔE*_00_ in Distilled Water	ΔE*_00_ in Black Tea	ΔE*_00_ in Instant Coffee	ΔE*_00_ in Red Wine
GC Cerasmart270	0.07	0.28	0.07	0.02
IPS e.max CAD	0.04	0.19	0.15	0.14
Telio CAD	0.13	0.03	0.01	0.01
Tetric CAD	0.07	0.23	0.02	0.08
Vita Enamic	0.04	0.09	0.12	0.01

**Table 14:** Difference in color change (ΔE*_00_) between black and white background—2 mm—after 4 weeks.

Material	ΔE*_00_ in Various Staining Solution			
	ΔE*_00_ in Distilled Water	ΔE*_00_ in Black Tea	ΔE*_00_ in Instant Coffee	ΔE*_00_ in Red Wine
GC Cerasmart270	0.09	0.38	0.01	0.02
IPS e.max CAD	−0.01	0.05	0.11	0.03
Telio CAD	0.05	0.01	0.01	0.10
Tetric CAD	−0.01	0.01	0.03	0.03
Vita Enamic	0.02	0.03	0.11	0.02

## Data Availability

Data is contained within the article.
